# The renal tubular damage marker urinary *N*-acetyl-β-d-glucosaminidase may be more closely associated with early detection of atherosclerosis than the glomerular damage marker albuminuria in patients with type 2 diabetes

**DOI:** 10.1186/s12933-017-0497-7

**Published:** 2017-01-26

**Authors:** So Ra Kim, Yong-ho Lee, Sang-Guk Lee, Eun Seok Kang, Bong-Soo Cha, Byung-Wan Lee

**Affiliations:** 1 Division of Endocrinology and Metabolism, Department of Internal Medicine, Graduate School, Yonsei University College of Medicine, 50-1, Yonsei-ro, Seodaemun-gu, Seoul, 03722 South Korea; 2 Severance Hospital, 50-1, Yonsei-ro, Seodaemun-gu, Seoul, 03722 South Korea; 3 Department of Laboratory Medicine, Yonsei University College of Medicine, 50-1, Yonsei-ro, Seodaemun-gu, Seoul, 03722 South Korea

**Keywords:** *N*-acetyl-β-d glucosaminidase, Carotid intima-media thickness, Carotid plaque, Type 2 diabetes mellitus

## Abstract

**Background:**

To determine the association between urinary *N*-acetyl-β-d-glucosaminidase (NAG), a marker of renal tubulopathy, and carotid intima-media thickness (IMT) and plaques in patients with type 2 diabetes mellitus (T2D) and to compare the predictive value of NAG versus albuminuria, a marker of renal glomerulopathy.

**Methods:**

A total of 343 participants were enrolled in this retrospective cross-sectional study. We recruited participants with T2D who were tested for blood glucose parameters, urinary NAG, and urinary albumin-to-creatinine ratio (ACR) and had been checked for carotid ultrasonography.

**Results:**

We classified participants into a below-median urinary NAG group (Group I; n = 172) or an above-median group (Group II; n = 171). Mean, maximum, and mean of maximum carotid IMT and the proportion of patients with carotid plaques were significantly higher in Group II compared with Group I. In multiple linear regression analyses, high urinary NAG (Group II) was significantly associated with carotid IMT, independently of urinary ACR and other confounding factors. In terms of carotid plaques, both urinary NAG and ACR were significantly higher in participants with carotid plaques than in those without carotid plaques. After adjustment for confounding factors, both urinary NAG and ACR were significantly associated with the presence of carotid plaques.

**Conclusions:**

Elevated urinary NAG, a marker of renal tubular damage, was related to increased carotid IMT and the presence of carotid plaques in patients with T2D. Urinary NAG may be a more sensitive biomarker than urinary albumin for early detection of atherosclerosis.

**Electronic supplementary material:**

The online version of this article (doi:10.1186/s12933-017-0497-7) contains supplementary material, which is available to authorized users.

## Background

Cardiovascular disease (CVD) is a major cause of morbidity and mortality in patients with type 2 diabetes mellitus (T2D), who have a twofold to fourfold increase in CVD incidence compared with patients without diabetes [1]. Therefore, the development of methods to estimate the risk of CVD in diabetic patients with more accuracy is highly desirable and will aid in preventing CVD events. Several surrogate measures for CVD that estimate subclinical atherosclerosis have been developed. Acceptable surrogates include pulse wave velocity (PWV), which is the gold standard for determining arterial stiffness and plays an important role in the occurrence of atherosclerosis [2, 3], carotid artery intima-media thickness (IMT) and the presence of carotid plaques, which, despite different pathogenic characteristics, correlate with coronary artery disease and stroke [4], and albuminuria, which is highly predictive not only of the development of diabetic kidney disease (DKD) but also of subsequent CVD and death [5]. Furthermore, several previous studies report positive independent associations between albuminuria and indices of macrovascular complications such as PWV and coronary artery calcification in patients with T2D or essential hypertension [6–8]. However, the relationship between IMT and albuminuria is still controversial [2, 9]. This controversy might be explained by differences in characteristics of study populations such as age, duration of diabetes, and ethnic diversity as well as the use of different methods to measure carotid atherosclerotic lesions [10].

Recently, several renal tubular damage markers have gained considerable attention because of their clinical relevance as sensitive and specific biomarkers for predicting the development and progression of early-stage DKD [11, 12]. The urinary enzyme *N*-acetyl-β-d-glucosaminidase (NAG) exists in the lysosomes of proximal tubule epithelial cells. Increased NAG excretion in urine is caused exclusively by proximal tubular cell injury. Furthermore, increases in urinary NAG already occur in normal to mildly increased albuminuric patients with T2D [11]. There is accumulating evidence that urinary NAG is correlated or associated not only with nephropathy but also with vascular complications of T2D, including retinopathy [13], neuropathy [14], and macrovascular disease [15]. However, little research has focused on the relationship between urinary NAG excretion and carotid IMT or plaques in patients with T2D.

The aim of this study was to investigate the association between urinary NAG and carotid IMT and plaques in patients with T2D. By comparing the predictive value of NAG with that of albuminuria, we also aimed to determine whether urinary NAG, a marker for renal tubulopathy, is a better predictor of carotid atherosclerosis than albuminuria, a marker for renal glomerulopathy.

## Methods

### Study participants

Using a retrospective cross-sectional design, we recruited participants with T2D who attended the Severance Hospital Diabetes Center between March 2015 and March 2016, had been simultaneously monitored for blood glucose parameters, urinary NAG, and albumin-to-creatinine ratio (ACR), and had undergone carotid ultrasonography within 1 month before or after the laboratory measurements. T2D was defined on the basis of (1) the participant’s use of insulin or oral hypoglycemic agents or (2) HbA_1C_ ≥ 6.5% (47.5 mmol/mol). We used the following inclusion criteria: (1) patients who had regular measurements of blood parameters including HbA_1C_ and (2) patients with stable HbA_1C_ indicated by changes of no more than 0.5% during the preceding 6 months. Participants who met the following criteria were excluded: (1) <20 years of age; (2) type 1 diabetes; (3) taking a sodium-glucose co-transporter 2 inhibitor; (4) pregnant women; (5) severe liver disease; (6) active cancer; (7) active infectious disease including urinary tract infection; (8) non-diabetic kidney disease; (9) connective tissue disease; (10) valvular heart disease or arrhythmia; (11) anemia (hemoglobin < 12 g/dL); or (12) hyper- or hypothyroidism. Age, sex, weight, height, waist circumference, smoking habits, blood pressure, duration of diabetes, and current medications were recorded. Body mass index (BMI) was calculated as weight divided by height squared (kg/m^2^). Hypertension was defined as a systolic blood pressure ≥140 mmHg and/or a diastolic blood pressure ≥90 mmHg or currently using antihypertensive medications. Coronary artery disease was defined as a positive history of myocardial infarction or proven single- or multi-vessel coronary artery disease with or without symptoms of angina pectoris. History of coronary artery disease or ischemic stroke had to be documented by hospital records. The Institutional Review Board at Severance Hospital approved this study protocol (4-2016-0480). Written informed consent for this study was not required by the Institutional Review Board because the database was only accessed for analysis purposes and personal information was not used.

### Measurements of blood gluco-metabolic parameters

Following an overnight fast, blood tests for glucose (designated as basal glucose), HbA_1C_, glycated albumin (GA), lipid profiles, white blood cell count, uric acid, and creatinine were performed. For measurements of postprandial glucose (designated as stimulated glucose), participants were administered a mixed-meal test, with blood samples collected 90 min after ingestion of two cans (total 400 mL, 400 kcal, 18 g fat, 44 g carbohydrate, and 20 g protein) of a standardized mixed-meal (Mediwell Diabetic MealTM, Meail Dairies Co., Yeongdong-gun, Chungbuk, Korea), or glucose was measured in blood collected 2 h after a normal meal. HbA_1c_ was measured by immunoassay using an Integra 800 CTS (Roche, Hercules, CA, USA). Serum GA levels were determined by an enzymatic method (LUCICA GA-L, Asahi Kasei Pharma Co., Tokyo, Japan) using a Hitachi 7600 auto-analyzer (Hitachi Ltd., Tokyo, Japan). Serum glucose and creatinine were also measured using the Hitachi 7600 analyzer (Hitachi Ltd.). For serum creatinine, a compensated kinetic Jaffe method (Clinimate CRE, Sekisui Medical Co., Ltd., Japan) was used, in which the creatinine concentration was standardized to isotope dilution-mass spectrometry. The estimated glomerular filtration rate (eGFR) was derived from the Chronic Kidney Disease Epidemiology Collaboration (CKD-EPI).

### Measurements of urinary glomerular and tubular damage markers

Urinary NAG, albumin, and creatinine levels were measured in a fasting morning spot urine sample that was obtained from each participant. Urinary NAG and albumin levels were expressed as urinary NAG-to-creatinine ratio and ACR to minimize the influence of variations in kidney function. The urine NAG level was measured by a colorimetric method using a reagent from Nittobo Medial Co., Ltd. (Tokyo, Japan) and a JCA-BM 6010/c automated chemistry analyzer (JEOL Ltd., Tokyo, Japan). In this assay, the substrate 6-methyl-2-pyridyl-*N*-acetyl-1-thio-β-d-glucosaminide is hydrolyzed by NAG and releases 6-methyl-2-pyridinethiol (MPT). The rate of increase of absorbance of MPT is measured at a wavelength of 340 nm to determine NAG activity in the sample. This assay is calibrated using a specific calibrator supplied by the reagent manufacturer with a lot-specific value of NAG activity. The urine albumin level was measured by an immunoturbidimetric method using an AU680 automated chemistry analyzer (Beckman Coulter, Inc., Brea, CA, USA). The urine creatinine level was also measured using the AU680 analyzer (Beckman Coulter, Inc.) by the kinetic Jaffe method.

### Measurements of carotid IMT

Protocols for measurements of IMT in both carotid arteries were described in detail previously [16]. Two specialized technicians conducted common carotid arterial ultrasound examinations using an Aloka ProSound ALPHA 10 (HITACHI, Tokyo, Japan) with a 13 MHz linear probe. IMT was defined as the distance between the media-adventitia interface and the lumen-intima interface. Mean IMT was defined as the average mean IMT of the right and left carotid arteries. Maximum IMT was the IMT value at the maximal point of the region, and the mean of maximum IMT was defined as the average of the maximum IMT values for both the right and left carotid arteries. Carotid plaques were defined according to the Mannheim consensus [17], in which a plaque is diagnosed when the vessel wall thickness is >1.5 mm or when the vessel wall appears to be at least 0.5 mm, or 50%, thicker than the surrounding wall. The presence of carotid plaques was positive when one or more carotid plaques existed.

### Statistical analyses

All statistical analyses were performed using SPSS version 20.0 for Windows (IBM Corp., Armonk, NY, USA). A normality test was performed for all continuous variables. The data are presented as mean ± standard deviation (SD) for normally distributed continuous variables and median (interquartile range) for non-normally distributed continuous variables. Categorical data are expressed as numbers and percentages. Urinary NAG-to-creatinine ratio was categorized into two groups: at or below the median NAG ratio (≤7.21 U/g creatinine) and above the median NAG ratio (>7.21 U/g creatinine). Categories of albuminuria were defined as follows: normal to mildly increased albuminuria (ACR < 30 mg/g creatinine) and moderate to severe albuminuria (ACR ≥ 30 mg/g creatinine). The characteristics of the study participants were analyzed depending on urinary NAG level or the presence of carotid plaques using Mann–Whitney U or two-sample Student’s t tests for continuous variables and a Pearson χ^2^ tests for categorical variables. Correlations between urinary NAG, urinary ACR, IMT, and other parameters were analyzed with Spearman’s correlation coefficients. Backward multiple linear regression analysis was performed on logarithm-transformed values of maximum or mean of maximum IMT in order to model the relationship between the IMT and demographic and laboratory parameters, including urinary NAG. Multiple logistic regression analyses were conducted to assess odds ratios (ORs) for predicting the presence of carotid plaques. In regression analyses, age was a binary variable defined as < or ≥40 years of age, which is when patients with T2D begin to be at high risk for CVD [18]. All P values <0.05 were considered statistically significant.

## Results

### Characteristics of the study participants depending on urinary NAG level

A total of 343 participants (203 men and 140 women) were enrolled in this study. The mean age and median duration of diabetes were 59.9 and 9.25 years, respectively. The median value of urinary NAG was 7.21 U/g creatinine. The participants were divided into two groups: at or below the median urinary NAG (Group I; urinary NAG, 4.89 (3.70–6.21) U/g creatinine) and above the median urinary NAG (Group II; urinary NAG, 11.4 (8.72–16.7) U/g creatinine). The demographics and laboratory characteristics of the participants are shown in Table 1. Age, duration of diabetes, and history of coronary artery disease were significantly higher in Group II than in Group I. Gender distribution, current smoking status, and history of hypertension or ischemic stroke were similar between the two groups. There were no significant differences in the proportions of participants taking glucose-lowering drugs, lipid-lowering drugs, angiotensin-converting-enzyme (ACE) inhibitors, angiotensin receptor blockers (ARBs), diuretics, calcium channel blockers, or beta blockers between the groups, but participants receiving antiplatelet or anticoagulant agents were more frequent in Group II than in Group I (Additional file 1: Table S1).

**Table 1:** Baseline demographics and laboratory characteristics of participants

Baseline characteristics	Total (N = 343)	Group I At or below median^a^ NAG (N = 172)	Group II Above median NAG (N = 171)	p values
Demographics				
Age (years)	59.9 ± 11.7	***57.2*** ***±*** ***11.7***	***62.5*** ***±*** ***11.0***	***<0.001***
Male sex [*n* (%)]	203 (59.2)	101 (58.7)	102 (59.6)	0.86
BMI (kg/m^2^)	25.1 (23.1–27.2)	24.8 (23.1–27.1)	25.3 (23.0–27.3)	0.32
Waist circumference (cm)	87.8 ± 8.95	86.9 ± 8.90	88.7 ± 8.98	0.07
Currently smoking [*n* (%)]	53 (15.5)	30 (17.4)	23 (13.5)	0.31
Hypertension [*n* (%)]	208 (60.6)	97 (56.4)	111 (64.9)	0.14
Systolic blood pressure (mmHg)	125.0 (116.0–133.0)	125.0 (115.0–132.3)	124.0 (117.0–133.0)	0.54
Diastolic blood pressure (mmHg)	74.4 ± 11.0	74.4 ± 11.2	74.6 ± 10.6	0.89
Coronary artery disease [*n* (%)]	89 (25.9)	***35 (20.3)***	***54 (31.6)***	***0.02***
Ischemic stroke [*n* (%)]	30 (8.75)	16 (9.30)	14 (8.19)	0.72
Duration of diabetes (years)	9.25 (5.25–16.3)	***7.25 (4.42***–***13.3)***	***11.3 (6.25***–***17.3)***	***0.001***
Laboratory indices				
HbA_1C_ (%)	6.80 (6.40–7.40)	***6.70 (6.40***–***7.10)***	***7.00 (6.50***–***7.70)***	***0.001***
Glycated albumin (%)	17.1 (15.7–19.5)	***16.6 (15.3***–***18.6)***	***18.1 (16.0***–***21.7)***	***<0.001***
Basal glucose (mg/dl)	128.0 (111.0–146.0)	***124.0 (110.0***–***140.0)***	***131.0 (114.0***–***156.3)***	***0.001***
Stimulated glucose (mg/dl)	191.5 (154.8–236.3)	***176.5 (140.3***–***214.8)***	***208.5 (170.8***–***255.8)***	***<0.001***
Total cholesterol (mg/dl)	156.0 (131.0–184.0)	155.0 (130.0–185.0)	157.5 (136.3–183.0)	0.48
Triglyceride (mg/dl)	116.0 (80.0–161.0)	115.0 (78.0–157.0)	118.5 (85.8–175.0)	0.28
HDL cholesterol (mg/dl)	44.0 (39.0–53.0)	44.0 (40.0–52.0)	42.0 (38.0–53.0)	0.17
LDL cholesterol (mg/dl)	83.6 (61.3–106.7)	81.6 (59.8–107.0)	84.6 (63.7–102.3)	0.90
White blood cell count (10^3^/µl)	6.46 (5.38–8.03)	6.41 (5.47–7.63)	6.55 (5.36–8.43)	0.50
Uric acid (mg/dl)	5.00 (4.20–5.90)	5.30 (4.30–5.90)	4.90 (4.00–5.93)	0.16
Creatinine (mg/dl)	0.79 (0.66–0.93)	0.77 (0.66–0.89)	0.82 (0.66–0.97)	0.12
eGFR CKD-EPI (ml/min/1.73 m^2^)	94.0 (82.0–101.0)	***96.0 (88.0***–***104.0)***	***91.0 (78.0***–***99.0)***	***<0.001***
Indices of diabetes complications				
Urinary NAG (U/g creatinine)	7.21 (4.89–11.4)	***4.89 (3.70***–***6.21)***	***11.4 (8.72***–***16.7)***	***<0.001***
Urinary ACR (mg/g creatinine)	10.0 (5.58–22.6)	***8.43 (4.90***–***17.8)***	***12.8 (6.54***–***34.6)***	***<0.001***
Mean carotid IMT (mm)	0.69 (0.60–0.80)	***0.67 (0.58***–***0.77)***	***0.72 (0.60***–***0.84)***	***0.02***
Maximum carotid IMT (mm)	0.85 (0.73–1.01)	***0.82 (0.70***–***0.97)***	***0.90 (0.74***–***1.06)***	***0.01***
Mean of maximum carotid IMT (mm)	0.81 (0.69–0.94)	***0.78 (0.66***–***0.91)***	***0.83 (0.71***–***0.98)***	***0.01***
Presence of carotid plaques [*n* (%)]	248 (72.3)	***114 (66.3)***	***136 (79.5)***	***0.01***

Continuous variables are described as mean ± SD for parametric variables and median (interquartile range) for nonparametric variables

*NAG N*-acetyl-β-d-glucosaminidase, *BMI* body mass index, *HDL* high density lipoprotein, *LDL* low density lipoprotein, *eGFR* estimated glomerular filtration rate, *CKD-EPI* chronic kidney disease epidemiology collaboration, *ACR* albumin-to-creatinine ratio, *IMT* intima-media thickness, *SD* standard deviation

Bolditalics denotes statistical significance at p < 0.05

^a^Median of NAG = 7.21 U/g creatinine

With respect to laboratory indices, the participants in Group II had significantly higher levels of blood glucose [HbA_1C_, GA, and basal and stimulated (postprandial) glucose] than those in Group I. Lipid profiles and serum creatinine did not significantly differ between the groups, but eGFR values calculated by the CKD-EPI equation were significantly lower in Group II than in Group I.

Regarding indices of diabetes complications, urinary ACR was significantly increased in Group II compared with Group I. Mean, maximum, and mean of maximum carotid IMT and the proportion of patients with carotid plaques were significantly higher in Group II than in Group I.

### Characteristics of the study participants depending on the presence of carotid plaques

The characteristics of the study participants depending on the presence of carotid plaques are shown in Additional file 2: Table S2. Compared with participants without carotid plaques, those with carotid plaques had higher age, longer duration of diabetes, and higher incidences of hypertension, coronary artery disease, and ischemic stroke. Regarding laboratory indices, the levels of total cholesterol and low density lipoprotein (LDL) cholesterol were significantly lower in participants with carotid plaques, which was consistent with their greater likelihood of taking lipid-lowering agents. Also, eGFR CKD-EPI was significantly lower in participants with carotid plaques compared with those without carotid plaques. Both urinary NAG and ACR were significantly higher in participants with carotid plaques than in those without carotid plaques. Antiplatelet or anticoagulant agents, lipid-lowering agents, ACE inhibitors or ARBs, calcium channel blockers, and beta blockers were more frequently used in participants with carotid plaques compared with those without carotid plaques.

### Correlations between urinary NAG and carotid IMT and other parameters

**Fig. 1:**
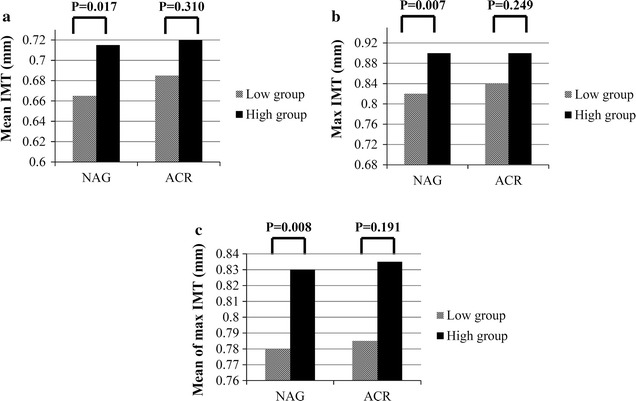
**a** Mean carotid IMT difference between high and low levels of urinary NAG or urinary ACR. **b** Maximum carotid IMT difference between high and low levels of urinary NAG or urinary ACR. **c** Mean of maximum carotid IMT difference between high and low levels of urinary NAG or urinary ACR (N = 343).* IMT* intima-media thickness,* NAG* *N*-acetyl-β-d-glucosaminidase,* ACR* albumin-to-creatinine ratio.* Each bar* represents the median. Significance was tested using Mann-Whitney U tests. Urinary NAG: low group, ≤7.21 U/g creatinine^a^; high group, >7.21 U/g creatinine. Urinary ACR: low group, <30 mg/g creatinine; high group, ≥30 mg/g creatinine. ^a^Median value of urinary NAG

Mean, maximum, and mean of maximum carotid IMT were positively correlated with age and waist circumference and negatively correlated with diastolic blood pressure, high density lipoprotein (HDL) cholesterol, and eGFR CKD-EPI (Table 2). However, measures of carotid IMT were not significantly correlated with duration of diabetes or blood glycemic parameters. Urinary NAG, measured as a continuous variable, was positively correlated with maximum carotid IMT and mean of maximum carotid IMT. When categorized into quartiles, urinary NAG was positively correlated with all measures of carotid IMT. However, urinary ACR was not significantly correlated with measures of carotid IMT, either as a continuous variable or by quartile. The mean, maximum, and mean of maximum carotid IMT were higher in the high urinary NAG group compared with the low urinary NAG group, whereas these differences were not found for urinary ACR (Fig. 1). When increased carotid IMT was defined as an IMT of >1 mm [19], the sensitivity of urinary NAG (>7.21 U/g creatinine; median value) and urinary ACR (≥30 mg/g creatinine) to identify an increased mean of maximum carotid IMT was 64.9 and 31.6%, respectively.

**Table 2:** Correlations between urinary NAG and carotid IMT and other parameters in T2D (N = 343)

Baseline characteristics	Urinary NAG		Mean IMT		Maximum IMT		Mean of maximum IMT	
	*r*	p	*r*	p	*r*	p	*r*	p
Demographics								
Age (years)	***0.27***	***<0.001***	***0.47***	***<0.001***	***0.44***	***<0.001***	***0.45***	***<0.001***
BMI (kg/m^2^)	0.03	0.54	0.08	0.16	0.10	0.06	0.10	0.07
Waist circumference (cm)	0.09	0.11	***0.13***	***0.01***	***0.17***	***0.001***	***0.17***	***0.002***
Systolic blood pressure (mmHg)	0.03	0.59	0.09	0.10	0.10	0.06	***0.11***	***0.04***
Diastolic blood pressure (mmHg)	0.002	0.97	***−0.24***	***<0.001***	***−0.23***	***<0.001***	***−0.22***	***<0.001***
Duration of diabetes (years)	***0.20***	***<0.001***	0.11	0.05	0.10	0.06	0.10	0.06
Laboratory indices								
HbA_1C_ (%)	***0.21***	***<0.001***	**−**0.07	0.19	**−**0.04	0.48	**−**0.05	0.36
Glycated albumin (%)	***0.27***	***<0.001***	0.03	0.61	0.04	0.50	0.04	0.52
Basal glucose (mg/dl)	***0.20***	***<0.001***	**−**0.10	0.06	**−**0.06	0.29	**−**0.07	0.22
Stimulated glucose (mg/dl)	***0.32***	***<0.001***	0.02	0.70	0.07	0.19	0.06	0.30
Total cholesterol (mg/dl)	0.03	0.56	**−**0.04	0.43	**−**0.02	0.71	**−**0.03	0.58
Triglyceride (mg/dl)	0.04	0.50	**−**0.04	0.42	**−**0.02	0.77	**−**0.03	0.54
HDL cholesterol (mg/dl)	***−0.14***	***0.01***	***−0.16***	***0.002***	***−0.15***	***0.01***	***−0.16***	***0.002***
LDL cholesterol (mg/dl)	0.02	0.74	0.04	0.48	0.04	0.51	0.04	0.50
White blood cell count (10^3^/µl)	0.03	0.70	0.04	0.64	0.12	0.16	0.07	0.44
Uric acid (mg/dl)	***−0.12***	***0.03***	0.08	0.14	0.10	0.07	0.09	0.08
eGFR CKD-EPI (ml/min/1.73 m^2^)	***−0.22***	***<0.001***	***−0.31***	***<0.001***	***−0.31***	***<0.001***	***−0.30***	***<0.001***
Indices of diabetes complications								
Urinary NAG (U/g creatinine)	1	–	0.10	0.07	***0.11***	***0.04***	***0.11***	***0.05***
Urinary NAG, quartile	–	–	***0.12***	***0.03***	***0.13***	***0.01***	***0.13***	***0.02***
Urinary ACR (mg/g creatinine)	***0.24***	***<0.001***	0.07	0.19	0.07	0.22	0.08	0.16
Urinary ACR, quartile	***0.23***	***<0.001***	0.05	0.36	0.04	0.48	0.05	0.33
Mean carotid IMT (mm)	0.10	0.07	1	–	***0.93***	***<0.001***	***0.97***	***<0.001***
Maximum carotid IMT (mm)	***0.11***	***0.04***	***0.93***	***<0.001***	1	–	***0.97***	***<0.001***
Mean of maximum carotid IMT (mm)	***0.11***	***0.05***	***0.97***	***<0.001***	***0.97***	***<0.001***	1	–

*NAG N*-acetyl-β-d-glucosaminidase, *IMT* intima-media thickness, *T2D* type 2 diabetes mellitus, *BMI* body mass index, *HDL* high density lipoprotein, *LDL* low density lipoprotein, *eGFR* estimated glomerular filtration rate, *CKD-EPI* chronic kidney disease epidemiology collaboration, *ACR* albumin-to-creatinine ratio

Bolditalics denotes statistical significance at p < 0.05

### Multiple linear regression analysis of factors affecting carotid IMT

To determine which factors predict carotid IMT, we performed multiple linear regression analyses (Table 3). Independent factors included in the model were age, eGFR CKD-EPI, urinary NAG, and urinary ACR, which were binary variables defined as <40 or ≥40 years, <60 or ≥60 ml/min/1.73 m^2^, ≤7.21 or >7.21 U/g creatinine, and <30 or ≥30 mg/g creatinine, respectively. We tested three models separately. In Model 1, we entered demographic parameters including age, sex, waist circumference, current smoking status, history of hypertension, and urinary NAG as independent factors and log transformed maximum or mean of maximum IMT as a dependent factor. Age, male gender, history of hypertension, and urinary NAG were significantly associated with both maximum and mean of maximum IMT. In Model 2, we additionally adjusted for HDL cholesterol and urinary ACR. Age, history of hypertension, and urinary NAG were significantly associated with maximum IMT, and age, history of hypertension, HDL cholesterol, and urinary NAG were significantly associated with mean of maximum IMT. Finally, further adjustment for eGFR CKD-EPI did not change the trends (Model 3).

**Table 3:** Multiple linear regression models for carotid IMT in T2D (N = 343)

Variables	Model 1		Model 2		Model 3	
	STD β	p	STD β	p	STD β	p
Log maximum IMT						
Age^a^	***0.19***	***0.003***	***0.19***	***0.003***	***0.19***	***0.003***
Male gender	***0.12***	***0.045***				
Waist circumference (cm)						
Currently smoking						
History of hypertension	***0.15***	***0.02***	***0.14***	***0.03***	***0.14***	***0.03***
HDL cholesterol (mg/dl)	–	–				
eGFR CKD-EPI^b^	–	–	–	–		
Urinary NAG^c^	***0.14***	***0.03***	***0.14***	***0.02***	***0.14***	***0.02***
Urinary ACR^d^	–	–				
Log mean of maximum IMT						
Age^a^	***0.19***	***0.003***	***0.18***	***0.004***	***0.18***	***0.004***
Male gender	***0.12***	***0.04***				
Waist circumference (cm)						
Currently smoking						
History of hypertension	***0.13***	***0.04***	***0.13***	***0.04***	***0.13***	***0.04***
HDL cholesterol (mg/dl)	–	–	***−0.13***	***0.03***	***−0.13***	***0.03***
eGFR CKD-EPI^b^	–	–	–	–		
Urinary NAG^c^	***0.13***	***0.03***	***0.13***	***0.03***	***0.13***	***0.03***
Urinary ACR^d^	–	–				

Logarithm-transformed values of maximum and mean of maximum carotid IMT were used for analysis

Model 1: adjusted for age, sex, waist circumference, current smoking status, history of hypertension, and urinary NAG

Model 2: adjusted for Model 1 + HDL cholesterol and urinary ACR

Model 3: adjusted for Model 2 + eGFR CKD-EPI

*IMT* intima-media thickness, *T2D* type 2 diabetes mellitus, *STD* standardized; HDL: high density lipoprotein, *eGFR*: estimated glomerular filtration rate, *CKD-EPI* chronic kidney disease epidemiology collaboration, *NAG N*-acetyl-β-d-glucosaminidase; *ACR* albumin-to-creatinine ratio

Bolditalics denotes statistical significance at p < 0.05

^a^Age, ^b^ eGFR CKD-EPI, ^c^ urinary NAG, and ^d^ urinary ACR were analyzed as binary variables defined as < or ≥40 years, < or ≥60 ml/min/1.73 m^2^, ≤ or >7.21 U/g creatinine (median of NAG = 7.21 U/g creatinine), and < or ≥30 mg/g creatinine, respectively

### Odds ratios for the presence of carotid plaques depending on urinary NAG and other risk variables

We performed multiple logistic regression analyses to investigate the association between carotid plaques and related risk factors, including urinary NAG, in participants without history of coronary artery disease or ischemic stroke (Table 4). In our unadjusted model (Model 1), an above- median level of urinary NAG (OR 1.96, 95% confidence interval (CI) 1.12–3.41, p = 0.02) was significantly associated with the presence of carotid plaques. However, urinary ACR level (OR 2.33, 95% CI 0.97–5.63, p = 0.06) was not significantly related to the presence of carotid plaques. After adjustment for age, sex, and history of hypertension, both urinary NAG and urinary ACR were significantly associated with the presence of carotid plaques (Model 2). After further adjustment for use of lipid-lowering drugs and eGFR CKD-EPI, the associations between urinary NAG or urinary ACR and carotid plaques remained significant (Model 3).

**Table 4:** Odds ratios for the presence of carotid plaques by urinary NAG and urinary ACR in participants without history of coronary artery disease or ischemic stroke (N = 232)

	ORs (95% CI)	
	Urinary NAG (0 = at or below median^a^, 1 = above median)	Urinary ACR (0 = normoalbuminuria^b^, 1 = micro- or macroalbuminuria^c^)
Model 1	***1.96 (1.12–3.41)***	2.33 (0.97–5.63)
Model 2	***1.88 (1.05–3.36)***	***3.42 (1.20–9.77)***
Model 3	***2.03 (1.12–3.68)***	***3.58 (1.25–10.3)***

Presence of carotid plaques was defined as the existence of one or more carotid plaques

Model 1: unadjusted

Model 2: adjusted for age (< or ≥40 years), sex, and history of hypertension

Model 3: adjusted for age (< or ≥40 years), sex, history of hypertension, use of lipid-lowering drugs, and eGFR CKD-EPI (< or ≥60 ml/min/1.73 m^2^)

*NAG N*-acetyl-β-d-glucosaminidase, *ACR* albumin-to-creatinine ratio, *ORs* odds ratios, *eGFR* estimated glomerular filtration rate, *CKD-EPI* chronic kidney disease epidemiology collaboration

Bolditalics denotes statistical significance at p < 0.05

^a^Median of NAG = 7.21 U/g creatinine, ^b^ ACR < 30 mg/g creatinine, ^c^ ACR ≥ 30 mg/g creatinine

## Discussion

Renal tubular damage markers, including urinary NAG, have gained considerable attention because of their clinical relevance as sensitive and specific biomarkers for predicting the development and progression of early-stage DKD [11, 12]. Furthermore, there is accumulating evidence that urinary NAG is correlated or associated with vascular complications of T2D, including not only nephropathy but also retinopathy [13], neuropathy [14], and macrovascular disease [15]. However, there is no information on the relationship between urinary NAG excretion and carotid atherosclerosis in patients with T2D. To our knowledge, only one study in patients with untreated hypertension has shown that urinary NAG excretion is positively associated with carotid IMT [20]. The aim of our study was to examine whether urinary NAG, a marker for renal tubulopathy, is more closely associated with carotid IMT and plaques in patients with T2D than albuminuria, a marker for renal glomerulopathy. In this study, we demonstrated several important findings. First, regarding carotid IMT, mean, maximum, and mean of maximum carotid IMT were significantly higher in the high NAG group than the low NAG group. In correlation analyses, urinary NAG showed a significant positive correlation with carotid IMT, whereas urinary ACR did not correlate significantly with carotid IMT. After adjustment for clinical and laboratory risk variables, including age, sex, waist circumference, current smoking status, hypertension, HDL cholesterol, eGFR, and urinary ACR, urinary NAG independently predicted measures of carotid IMT. Second, in terms of carotid plaques, both urinary NAG and ACR were significantly higher in participants with carotid plaques than in those without carotid plaques. In addition, having an above-median level of urinary NAG was associated with approximately twice the risk for the presence of carotid plaques compared with urinary NAG levels at or below the median. After adjustment for confounding variables, both urinary NAG and urinary ACR were significantly associated with the presence of carotid plaques.

Recently, attention to renal tubular damage markers as potential risk factor indicators for CVD is growing. There is accumulating evidence that urinary NAG is associated with various vascular diseases in different populations. In the GISSI-Prevenzione trial targeting subjects with chronic heart failure [21], urinary NAG level was consistently and strongly related to poor outcomes. In studies of type 1 or type 2 diabetes, retinopathy and peripheral neuropathy correlated with urinary NAG [13, 14, 22]. Weitgasser et al. [15] showed the association of urinary NAG with macrovascular disease in elderly T2D patients. That study investigated patients with T2D during a median follow-up of 7 years and showed that urinary NAG is comparable to albuminuria as an indicator of the preexistence and development of severe macrovascular disease, including myocardial infarction and peripheral vascular disease. Regarding subjects without diabetes, Ouchi et al. [23] reported that elevated urinary NAG is related to elevated arterial stiffness assessed by brachial-ankle PWV. In a study including a low-risk general population, urinary NAG and ACR were independently associated with first-ever myocardial infarction, first-ever ischemic stroke, and all-cause mortality. However, NAG did not add to the risk predicted by traditional cardiovascular risk factors, such as eGFR and ACR [24].

With respect to the relationship between albuminuria as a glomerular damage marker and subclinical atherosclerosis as assessed by IMT, although the association of albuminuria with CVD has been well-established in a number of populations, including diabetics, hypertensive patients, and general subjects [25, 26], the relationship between carotid IMT and albuminuria is still controversial, as some studies show conflicting results. Among studies of patients with T2D, Ishimura et al. [2] and Ito et al. [27] reported that the significant association between these two parameters was lost after adjustment for traditional risk factors such as blood pressure and waist-hip ratio. There may be several explanations for this discrepancy. First, diabetes itself has such a powerful influence on carotid IMT that any effect of albuminuria could be masked [9]. Second, the study populations differed in terms of age, duration of diabetes, eGFR, ethnicity, and the use of methods of measuring carotid atherosclerotic lesions [27].

Only a single study, to our knowledge, has investigated the association between subclinical atherosclerosis of IMT and urinary NAG [20]. It showed that urinary NAG and carotid IMT or PWV were positively correlated and that carotid IMT and PWV had significant positive effects on urinary NAG in regression analyses of patients with untreated hypertension. In the present study, we observed that measures of carotid IMT were significantly associated with urinary NAG rather than with urinary ACR in simple correlation and regression analyses. Because of the cross-sectional design of our study, possible mechanisms for the relationship between urinary NAG and carotid IMT remain unclear. There is evidence that chronic tubulointerstitial hypoxia and increased oxidative stress may play a pathogenic role in early stages of DKD [28], which in turn is related to the development of CVD. Furthermore, increased urinary NAG is a sensitive marker of tubular injury related to inflammation and oxidative stress [24]. Therefore, the elevation of urinary NAG, a renal interstitial marker, might reflect the severity of interstitial changes that result from atherosclerosis and subsequent ischemic injury [23]. In addition, the association between urinary NAG and carotid IMT might be mediated by several common risk factors. Consistent with previous reports [29], we observed that both urinary NAG and measures of carotid IMT were positively correlated with age and negatively correlated with eGFR, which are well-known risk factors for both DKD and systemic atherosclerosis. Nevertheless, we found several differences in the correlations of urinary NAG versus measures of carotid IMT with metabolic variables, including glucose-homeostasis and blood pressure. We previously suggested that increased urinary NAG is related to glycemic parameters reflecting glucose fluctuation (e.g., postprandial glucose rather than fasting glucose, GA rather than HbA_1C_) and decreased insulin secretory capacity as assessed by HOMA-β or postprandial C-peptide-to-glucose ratio (PCGR) in patients with T2D [12]. Regarding plausible explanations for associations between glucose fluctuation and increases in urinary NAG excretion, we suggested the possibility of a physiologic increase in urinary NAG for metabolizing urinary glucose and a nephrotoxic effect of glycated end products on renal proximal tubules. NAG is an enzyme involved in carbohydrate metabolism; thus, when the proximal tubules are exposed to high urinary glucose, NAG might be more highly secreted in the urine, depending on urinary glucose concentrations [30]. In addition, the peptides derived from advanced glycation end products could have a nephrotoxic effect on the proximal tubule, thus contributing to the occurrence of proximal tubule injury [31]. We previously suggested [32] that serum GA is more closely associated with postprandial glucose than fasting glucose and might be a useful index for monitoring fluctuating and poorly controlled glycemic excursions. The clinical relevance of GA could be attributed to observations that increased GA rather than HbA_1C_ is significantly correlated with insulin secretory beta-cell function as assessed by PCGR and HOMA-β and that GA increases as the duration of diabetes lengthens [33]. Despite the lack of association between HbA_1C_ and IMT, consistent with previous studies [34], increasing waist circumference was associated only with measures of carotid IMT, which could be explained by the association of insulin resistance with increased IMT [35, 36]. However, neither urinary NAG nor measures of carotid IMT were significantly associated with BMI [37]. Urinary NAG was not related to high blood pressure, as was noted in a previous study [15], whereas measures of carotid IMT were negatively correlated with diastolic blood pressure.

There is a well-established association between urinary ACR and carotid plaques in patients with T2D [10, 38]. In this study, we found that the presence of carotid plaques was associated with age, long duration of diabetes, history of hypertension, coronary artery disease, or ischemic stroke, reduction in eGFR, and increases in both urinary ACR and urinary NAG. An above-median level of urinary NAG (OR 1.96, 95% CI 1.12–3.41, p = 0.02) was significantly associated with the presence of carotid plaques. However, urinary ACR levels (OR 2.33, 95% CI 0.97–5.63, p = 0.06) were not significantly related to the presence of carotid plaques in an unadjusted model. After adjustment for age, sex, and history of hypertension, both urinary NAG and urinary ACR were significantly associated with the presence of carotid plaques. After further adjustment to use lipid-lowering drugs and eGFR CKD-EPI, the associations between urinary NAG or urinary ACR and carotid plaques remained significant. These results show that urinary NAG is comparable to urinary ACR in predicting the presence of carotid plaques. To our knowledge, this is the first time the relationship between urinary NAG and carotid plaques has been investigated.

The current study has several strengths. First, our study provides clinical evidence supporting associations between urinary NAG and various vascular complications of diabetes. Second, we investigated whether urinary NAG, a renal tubulopathic marker, is more closely associated with carotid atherosclerosis than the glomerulopathic marker albuminuria, which might reinforce the comparability between markers. Additionally, we conducted the study with a relatively large number of subjects in a single center trial, which strengthens the statistical reliability of the results. However, the current study also has some limitations. First, because of the inherent drawbacks of cross-sectional studies, we cannot infer any causal or temporal relationships between urinary NAG and carotid atherosclerosis. Second, the values of Spearman’s correlation coefficients between urinary NAG and measures of carotid IMT were statistically significant but of only slight to fair magnitude. The process of increasing IMT is a complex phenomenon that is not only determined by pure atherosclerotic risk factors [39]. Thus, multifactorial determinants of IMT may reduce the statistical significance of urinary NAG. Finally, we did not compare the sensitivity of detecting carotid atherosclerosis of urinary NAG using other renal tubular damage markers. For example, urinary liver-type fatty acid binding protein is also a sensitive marker of renal injury [40].

## Conclusions

We evaluated the association of urinary NAG with subclinical atherosclerosis as assessed by carotid IMT and revealed a novel finding that increased carotid IMT and the presence of carotid plaques are related to elevated urinary NAG, a marker of renal tubular damage, in patients with T2D. Furthermore, we found that urinary NAG might be a more sensitive urinary biomarker than urinary albumin for early detection of atherosclerosis. Further longitudinal prospective studies are needed to investigate the causal relationship between renal tubular damage and systemic atherosclerosis and its mechanisms.

## Additional files

**Additional file 1: Table S1.** Baseline demographics and laboratory characteristics of participants.

**Additional file 2: Table S2.** Baseline demographics and laboratory characteristics of participants with and without carotid plaques (N = 343).

## Abbreviations

ACE: angiotensin-converting-enzyme
ACR: albumin-to-creatinine ratio
ARBs: angiotensin receptor blockers
BMI: body mass index
CI: confidence interval
CKD-EPI: chronic kidney disease epidemiology collaboration
CVD: cardiovascular disease
DKD: diabetic kidney disease
DPP-IV: dipeptidyl peptidase-IV
eGFR: estimated glomerular filtration rate
GA: glycated albumin
HDL: high density lipoprotein
IMT: intima-media thickness
LDL: low density lipoprotein
MPT: 6-methyl-2-pyridinethiol
NAG: *N*-acetyl-β-d-glucosaminidase
OR: odds ratio
PCGR: postprandial C-peptide-to-glucose ratio
PWV: pulse wave velocity
SD: standard deviation
T2D: type 2 diabetes mellitus
