# Supplementary material for: The renal tubular damage marker urinary N-acetyl-β-d-glucosaminidase may be more closely associated with early detection of atherosclerosis than the glomerular damage marker albuminuria in patients with type 2 diabetes
Source: Cardiovasc Diabetol. 2017 Jan 26;16:16. doi: 10.1186/s12933-017-0497-7 (PMC5267389; doi:10.1186/s12933-017-0497-7)
Supplement: Supplementary file 1 — Additional file 1: Table S1. Baseline demographics and laboratory characteristics of participants. [file 12933_2017_497_MOESM1_ESM.docx]

**Table S1.** Baseline demographics and laboratory characteristics of participants.

| Baseline characteristics |  | **Total (N = 343)** | **Group I**  **At or below median^a^**  **NAG (N = 172)** | **Group II**  **Above median**  **NAG (N = 171)** | **p**  **values** |
| --- | --- | --- | --- | --- | --- |
| **Medications** |  |  |  |  |  |
| Glucose-lowering drug-naïve [*n* (%)] |  | 7 (2.04) | 2 (1.16) | 5 (2.92) | 0.28 |
| Insulin [*n* (%)] |  | 57 (16.6) | 25 (14.5) | 32 (18.7) | 0.30 |
| Metformin [*n* (%)] |  | 303 (88.3) | 157 (91.3) | 146 (85.4) | 0.08 |
| DPP-IV inhibitor [*n* (%)] |  | 165 (48.1) | 78 (45.3) | 87 (50.9) | 0.33 |
| Thiazolidinediones [*n* (%)] |  | 38 (11.1) | 19 (11.0) | 19 (11.1) | 0.99 |
| Sulfonylurea [*n* (%)] |  | 127 (37.0) | 61 (35.5) | 66 (38.6) | 0.58 |
| Antiplatelet/anticoagulant agents [*n* (%)] |  | 158 (46.1) | **70 (40.7)** | **88 (51.5)** | **0.045** |
| Lipid lowering drugs [*n* (%)] |  | 197 (57.4) | 104 (60.5) | 93 (54.4) | 0.25 |
| ACEi/ARB [*n* (%)] |  | 130 (37.9) | 61 (35.5) | 69 (40.4) | 0.35 |
| Diuretics [*n* (%)] |  | 23 (6.71) | 10 (5.81) | 13 (7.60) | 0.51 |
| Calcium channel blockers [*n* (%)] |  | 76 (22.2) | 34 (19.8) | 42 (24.6) | 0.29 |
| Beta blockers [*n* (%)] |  | 61 (17.8) | 27 (15.7) | 34 (19.9) | 0.31 |

NAG: N-acetyl-β-D-glucosaminidase; DPP-IV: dipeptidyl peptidase-IV; ACEi: angiotensin-converting-enzyme inhibitor; ARB: angiotensin receptor blocker.

^a^Median of NAG = 7.21 U/g creatinine.

Bold denotes statistical significance at p <0.05.
