# Supplementary material for: The renal tubular damage marker urinary N-acetyl-β-d-glucosaminidase may be more closely associated with early detection of atherosclerosis than the glomerular damage marker albuminuria in patients with type 2 diabetes
Source: Cardiovasc Diabetol. 2017 Jan 26;16:16. doi: 10.1186/s12933-017-0497-7 (PMC5267389; doi:10.1186/s12933-017-0497-7)
Supplement: Supplementary file 2 — Additional file 2: Table S2. Baseline demographics and laboratory characteristics of participants with and without carotid plaques (N = 343). [file 12933_2017_497_MOESM2_ESM.docx]

**Table S2.** Baseline demographics and laboratory characteristics of participants with and without carotid plaques (N = 343).

|  | |  | **Carotid Plaques** | | |
| --- | --- | --- | --- | --- | --- |
| Baseline characteristics |  |  | **No (N = 93)** | **Yes^a^ (N = 250)** | **p values** |
| **Demographics** |  |  |  |  |  |
| Age (years) |  |  | **52.1 ± 11.9** | **62.8 ± 10.1** | **<0.001** |
| Male Sex [*n* (%)] | |  | 49 (52.7) | 156 (62.4) | 0.10 |
| BMI (kg/m^2^) | |  | 25.2 (23.2-27.7) | 25.0 (23.0-27.1) | 0.49 |
| Waist circumference (cm) | |  | 87.0 ± 9.82 | 88.1 ± 8.61 | 0.31 |
| Currently smoking [*n* (%)] | |  | 17 (18.3) | 36 (14.4) | 0.39 |
| Hypertension [*n* (%)] | |  | **42 (45.2)** | **168 (67.2)** | **<0.001** |
| Systolic blood pressure (mmHg) | | 124.0 (114.0-132.0) | | 125.0 (117.0-133.0) | 0.28 |
| Diastolic blood pressure (mmHg) | |  | **76.5 ± 10.5** | **73.7 ± 11.1** | **0.04** |
| Coronary artery disease [*n* (%)] | |  | **9 (9.70)** | **80 (32.0)** | **<0.001** |
| Ischemic stroke [*n* (%)] | |  | **2 (2.20)** | **29 (11.6)** | **0.01** |
| Duration of diabetes (years) | |  | **7.25 (4.25-12.3)** | **10.3 (5.54-17.3)** | **<0.001** |
| **Laboratory indices** | |  |  |  |  |
| HbA_1C_ (%) | |  | 6.85 (6.33-7.40) | 6.80 (6.40-7.45) | 0.72 |
| Glycated albumin (%) | |  | 17.1 (15.7-19.7) | 17.1 (15.7-19.5) | 0.99 |
| Basal glucose (mg/dl) | | 129.0 (113.0-152.0) | | 126.5 (110.8-145.0) | 0.30 |
| Stimulated glucose (mg/dl) |  | 186.0 (148.0-234.0) | | 194.0 (159.0-237.0) | 0.37 |
| Total cholesterol (mg/dl) |  | **162.0 (139.5-192.5)** | | **155.0 (130.0-180.3)** | **0.03** |
| Triglyceride (mg/dl) |  |  | 117.0 (76.5-156.5) | 115.5 (85.8-162.0) | 0.31 |
| HDL cholesterol (mg/dl) |  |  | 44.0 (39.5-53.5) | 43.0 (38.0-52.3) | 0.13 |
| LDL cholesterol (mg/dl) |  |  | **91.8 (68.6-109.5)** | **81.6 (59.8-102.3)** | **0.02** |
| White blood cell count (10^3^/µl) | |  | 6.46 (5.39-7.85) | 6.46 (5.36-8.15) | 0.67 |
| Uric acid (mg/dl) |  |  | 5.00 (4.20-5.80) | 5.00 (4.20-6.00) | 0.71 |
| Creatinine (mg/dl) |  |  | 0.75 (0.64-0.90) | 0.80 (0.67-0.95) | 0.09 |
| eGFR CKD-EPI (ml/min/1.73 m^2^) | |  | **97.0 (91.0-107.0)** | **92.0 (79.0-100.0)** | **<0.001** |
| **Indices of diabetes complications** | |  |  |  |  |
| Urinary NAG (U/g creatinine) | |  | **6.35 (4.40-8.35)** | **7.53 (5.24-12.0)** | **0.003** |
| Urinary ACR (mg/g creatinine) | |  | **7.52 (4.76-13.1)** | **11.5 (6.17-27.7)** | **<0.001** |
| Mean carotid IMT (mm) | |  | **0.61 (0.55-0.71)** | **0.73 (0.62-0.84)** | **<0.001** |
| Maximum carotid IMT (mm) | |  | **0.74 (0.66-0.85)** | **0.90 (0.77-1.05)** | **<0.001** |
| Mean of maximum carotid IMT (mm) | |  | **0.70 (0.63-0.82)** | **0.84 (0.72-0.99)** | **<0.001** |
| **Medications** | |  |  |  |  |
| Glucose-lowering drug-naïve [*n* (%)] | |  | 2 (2.20) | 5 (2.00) | >0.999 |
| Insulin [*n* (%)] | |  | 18 (19.4) | 40 (16.0) | 0.46 |
| Metformin [*n* (%)] | |  | 83 (89.2) | 222 (88.8) | 0.91 |
| DPP-IV inhibitor [*n* (%)] | |  | 40 (43.0) | 125 (50.0) | 0.25 |
| Thiazolidinediones [*n* (%)] | |  | 11 (11.8) | 28 (11.2) | 0.87 |
| Sulfonylurea [*n* (%)] | |  | 29 (31.2) | 98 (39.2) | 0.17 |
| Antiplatelet/anticoagulant agents [*n* (%)] | |  | **16 (17.2)** | **142 (56.8)** | **<0.001** |
| Lipid lowering drugs [*n* (%)] | |  | **42 (45.2)** | **156 (62.4)** | **0.004** |
| ACEi/ARB [*n* (%)] | |  | **27 (29.0)** | **105 (42.0)** | **0.03** |
| Diuretics [*n* (%)] | |  | 3 (3.20) | 21 (8.40) | 0.10 |
| Calcium channel blockers [*n* (%)] | |  | **10 (10.8)** | **66 (26.4)** | **0.002** |
| Beta blockers [*n* (%)] | |  | **7 (7.50)** | **54 (21.6)** | **0.002** |

BMI: body mass index; HDL: high density lipoprotein; LDL: low density lipoprotein; eGFR: estimated glomerular filtration rate; CKD-EPI: Chronic Kidney Disease Epidemiology Collaboration; NAG: N-acetyl-β-D-glucosaminidase; ACR: albumin-to-creatinine ratio; IMT: intima-media thickness; DPP-IV: Dipeptidyl peptidase-IV; ACEi: angiotensin-converting-enzyme inhibitor; ARB: angiotensin receptor blocker; SD: standard deviation.

Continuous variables are described as mean ± SD for parametric variables and median (interquartile range) for nonparametric variables.

^a^Presence of carotid plaques was defined as the existence of one or more carotid plaques.

Bold denotes statistical significance at p <0.05.
